# Comparison of Side Effects of Anti-epidermal Growth Factor Receptor (Anti-EGFR) Antibody Drugs During Initial Induction and Reinduction in Colorectal Cancer: A Case Series

**DOI:** 10.7759/cureus.87184

**Published:** 2025-07-02

**Authors:** Tadafumi Hoshida, Kazufumi Tanabe, Masanobu Tsubaki, Noriaki Nagai, Shozo Nishida

**Affiliations:** 1 Department of Pharmacy, Japanese Red Cross Wakayama Medical Center, Wakayama, JPN; 2 Laboratory of Pharmacotherapy, Kagawa School of Pharmaceutical Sciences, Tokushima Bunri University, Sanuki, JPN; 3 Department of Pharmacy, Kindai University, Higashi-osaka, JPN

**Keywords:** anti-egfr antibody drugs, chemotherapy, colorectal cancer, reinduction, side effects

## Abstract

Anti-epidermal growth factor receptor (anti-EGFR) antibodies are used to treat colorectal cancer. The incidence of side effects is higher in patients who receive re-administration than in those who were initially introduced. In this report, we describe three cases. Case 1 involved a woman in her 70s. Compared with the initial administration, skin rash, skin dryness, and stomatitis grades were observed to be high. Finally, the patient was diagnosed with progressive disease (PD) after five months, which was shorter than that at the time of initial administration. Case 2 involved a man in his 70s, who was restarted on panitumumab monotherapy. Approximately one month after re-administration, the patient developed early grade 3 skin symptoms and hypomagnesemia, peaking at 0.8 mg/dL. Finally, the patient was diagnosed with PD after approximately six months, which was shorter than that at the time of initial administration. Case 3 involved a man in his 30s. After resuming the same treatment, the patient developed paronychia up to grade 3, which continued for approximately two years with dermatological intervention. In the ambulatory therapy center, multi-disciplinary medical staff are involved in the prevention of serious complications. Early intervention and patient education regarding the adverse effects connected with re-administration are essential.

## Introduction

Various drug therapies have been used for cancer treatment, and the treatment results are improving. Compared to cases in which they could not be used adequately owing to side effects, to use skillfully three key drugs (fluoropyrimidines, oxaliplatin (L-OHP) , and irinotecan (CPT-11)) prolongs overall survival [1]. In particular, the mFOLFOX6 (5-fluorouracil (5-FU), folinic acid (l-LV), L-OHP) and FOLFIRI (5-FU, l-LV, CPT-11) regimens are widely used in clinical practice as first-line therapy. Recently, molecular-targeted drugs and immune checkpoint inhibitors have also gained indications and have been shown to prolong overall survival year after year [2].

Cancer is historically known to have monoclonality and heterogeneity within the tumor and has been considered a cause of resistance to treatment [3,4]. This causes cancer to proliferate indefinitely, and differences in the degree of proliferation result in areas within the tumor mass that are sensitive to anticancer drugs and regions that are not. While the number of drug options has increased, there have been reports of cases in which all drugs used in standard treatment have been exhausted, and there have been reports of further prolongation of progression-free survival and overall survival by the re-administration of drugs that have shown therapeutic efficacy in the past [5,6]. However, molecularly-targeted drugs are also known to cause side effects specific to their target molecules because of their mechanisms of action. few studies have evaluated the occurrence and frequency of side effects.

In this report, we describe three cases of patients who were re-administered an anti-epidermal growth factor receptor (anti-EGFR) antibody, a molecular target drug, and in whom the onset of side effects occurred earlier and more strongly after re-administration than after initial administration.

## Case presentation

We retrospectively reviewed the electronic medical records of patients who received the anti-EGFR antibody drugs, cetuximab (Cmab) and panitumumab (Pmab), between April 2011 and December 2020 at the Japanese Red Cross Wakayama Medical Center. The TNM classification was based on the physician’s evaluation of the Japanese Classification of Colorectal, Appendiceal, and Anal Carcinoma (8th edition) [7]. Treatment response was based on the Response Evaluation Criteria in Solid Tumors (RECIST) v1.1 [8]. Adverse events were evaluated based on the Japanese translation by the Japan Clinical Oncology Group (JCOG) of the Common Terminology Criteria for Adverse Events (CTCAE) v4.0 [9]. This case series was approved by the Ethical Review Committee of the Japanese Red Cross Wakayama Medical Center (approval number: 1164).　

Three cases are presented here. The backgrounds and the treatment results of the three patients are summarized in Table 1. In all three cases, a partial response (PR) was achieved at the time of initial introduction. Upon reintroduction, the same regimen was used in Cases 1 and 3 as at the time of initial administration. In Case 2, Pmab monotherapy was selected due to L-OHP allergy and poor general condition. Side effects occurred earlier and were stronger at the time of re-administration than at the initial administration.

**Table 1:** Patient background in the three cases 5-FU: 5-fluorouracil; L-OHP: oxaliplatin; CPT-11: irinotecan; l-LV: folinic acid; Cape: capecitabine; Pmab: paniutmumab; mFOLFOX6: 5-FU+l-LV+L-OHP; FOLFIRI: 5-FU+l-LV+CPT-11; CAPOX+Pmab: Cape+L-OHP+Pmab; PR: partial response; SD: stable disease; PD: progressive disease

	Case 1	Case 2	Case 3
Age	70s	70s	30s
Sex	Female	Male	Male
Location	Sigmoid	Sigmoid	Descending
Regimen (initial)	mFOLFOX6+Pmab	mFOLFOX6+Pmab→FOLFIRI+Pmab	CAPOX+Pmab
Best response	PR	PR	PR
Disease-free survival (days)	356	476	426
Regimen (reintroduction)	mFOLFOX6+Pmab	Pmab	CAPOX+Pmab
Best response	SD	SD	PD
Disease-free survival (days)	171	168	133

Case 1

**Figure 1:**
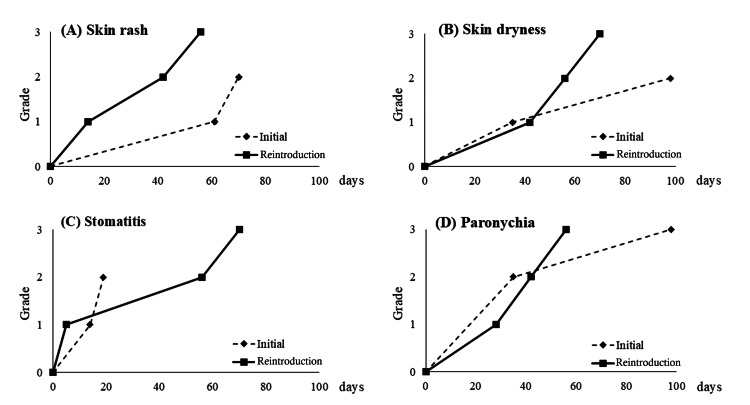
Transitions in side effects between inital administration and reintroduction of anti-EGFR antibody in Case 1 (A) Skin rash, (B) Skin dryness, (C) Stomatitis, (D) Paronychia The vertical axis shows the grade of each side effect, and the horizontal axis shows the number of days since the start of administration. Stomatitis first appeared. The grades were higher than those at the time of initial induction in all except for (D). EGFR: epidermal growth factor receptor

The patient, a woman in her 70s, had stage IV sigmoid colon cancer and underwent mFOLFOX6 + Pmab therapy as the primary treatment. After approximately six months of treatment, PR was achieved, and the primary tumor and liver metastases were resected. About five months later, the same therapy was resumed owing to early postoperative recurrence of the residual liver and new lung metastasis. On the re-administration date, hypomagnesemia and a visible range of skin symptoms resolved. Compared to initial administration, the skin rash, skin dryness, and stomatitis grades were higher, and the patient was forced to discontinue therapy due to side effects. Chemotherapy was suspended because of side effects, and treatment was resumed once the patient's condition improved. Finally, the patient was diagnosed with progressive disease (PD) after five months, which was shorter than that at the time of initial administration. Despite the relative dose intensity (RDI) being decreased from 84% to 58% during re-administration, the patient remarked, “The side effects are stronger than when I first underwent the therapy.” The change in side effects between initial induction and reinduction is shown in Figure 1.

Case 2

The patient, a man in his 70s, had stage IV sigmoid colon cancer and underwent mFOLFOX6 + Pmab therapy as the primary treatment. The patient developed an allergy during L-OHP administration in the third course; hence, the treatment was changed to the FOLFIRI + Pmab regimen. At this time, there was no worsening of gastrointestinal symptoms due to the change from L-OHP to CPT-11. In total, the Pmab regimen continued for approximately one year and three months until PD was achieved. After that, the patient underwent the FOLFIRI + bevacizumab (Bev) regimen as a second-line treatment for about six months. During second-line treatment, there was no worsening of side effects, and skin symptoms gradually improved.

**Figure 2:**
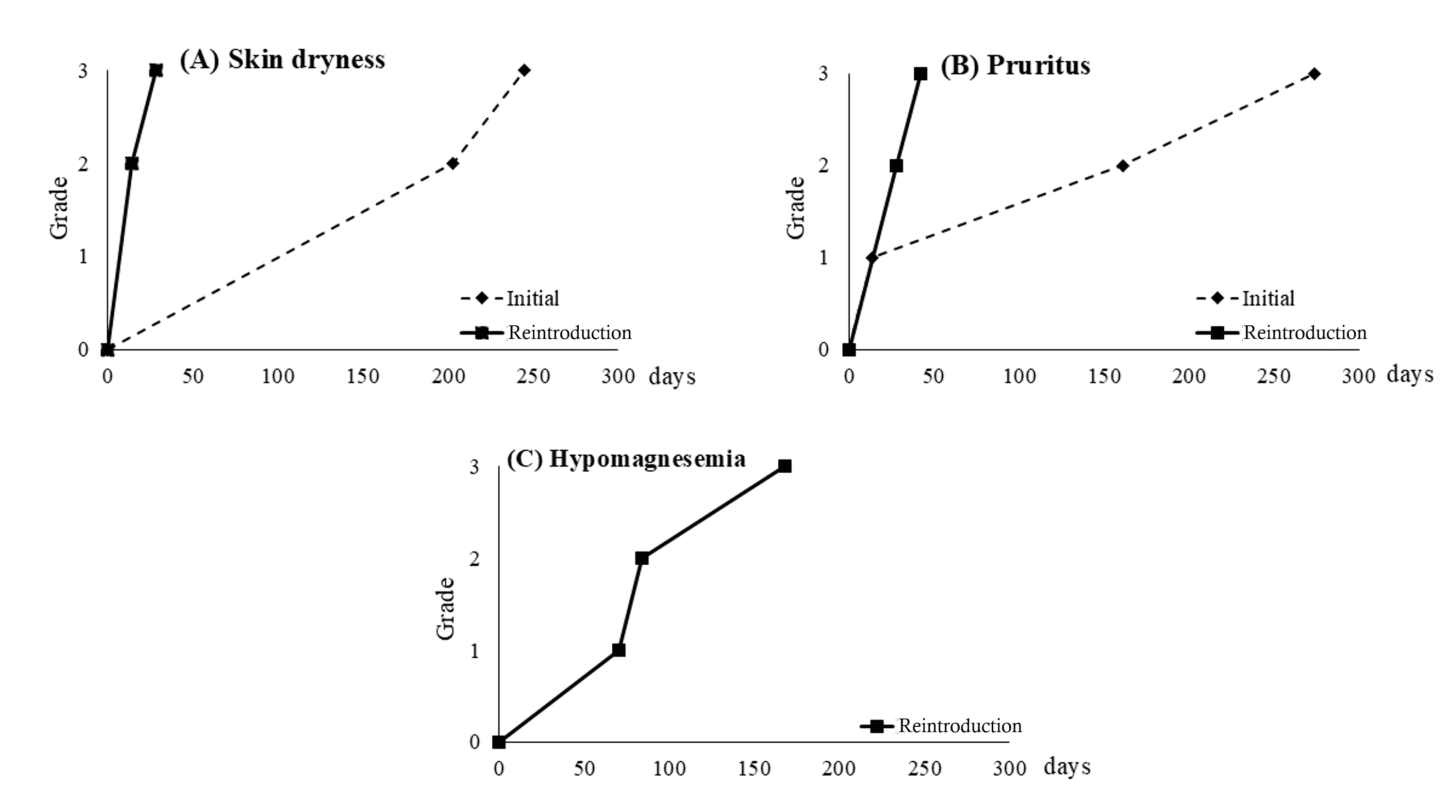
Transitions in side effects between inital administration and reintroduction of anti-EGFR antibody in Case 2 (A) Skin dryness, (B) Pruritus, (C) Hypomagnesemia* (A) and (B) reached grade 3 earlier at the time of reintroduction. In (C), improvement was poor despite withdrawal and magnesium supplementation. *Unlike other side effects, hypomagnesemia did not occur at the time of initial administration of anti-EGFR EGFR: epidermal growth factor receptor

The patient was reintroduced to Pmab monotherapy in the third regimen. At this point, the skin symptoms had disappeared. Nevertheless, early skin symptoms reached grade 3 approximately one month after re-administration. Also, the patient developed hypomagnesemia, which was not present at the time of initial administration, peaking at 0.8 mg/dL to grade 3. Although no abnormalities were found in the electrocardiogram test, the fatigue was also strong during this period, and together with skin symptoms. The patient remarked that it was “more painful than the initial administration.” Recovery was not sufficient with intravenous magnesium 20 mmol/L replenishment on chemotherapy days, so Pmab therapy was discontinued. Recovery was finally achieved after approximately one month with three magnesium replenishments per week. After that, Pmab therapy was resumed, and despite repeated skin symptoms, appetite loss, and dehydration, it was continued for about five months until PD while administering fluid replacement therapy. The change in side effects between initial induction and reinduction is shown in Figure 2.

Case 3

**Figure 3:**
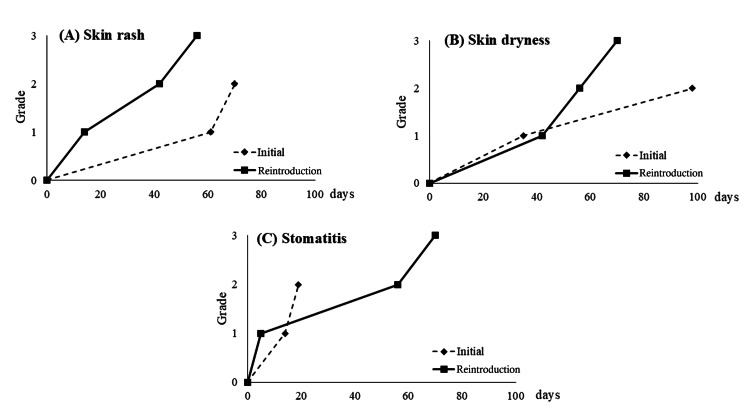
Transitions in side effects between inital administration and reintroduction of anti-EGFR antibody in Case 3 (A) Skin rash, (B) Skin dryness, (C) Stomatitis At the time of reintroduction, the patient achieved grade 3, which was higher than at the initial administration. EGFR: epidermal growth factor receptor

The patient, a male in his 30s, relapsed with descending colon cancer during postoperative adjuvant chemotherapy with tegafur/uracil (UFT) and folinate. Due to a busy work schedule, central venous port creation was not desired, and tegafur/gimeracil/oteracil (TS-1) monotherapy was started as a second-line. After approximately two years of TS-1 monotherapy, PD occurred, and capecitabine/L-OHP (CAPOX) + Pmab therapy was introduced as a third-line treatment. During this therapy, pulmonary congestion was accidentally observed on follow-up CT scans, and the patient was diagnosed with chronic heart failure. Colorectal cancer treatment was temporarily suspended for approximately three months to control heart failure and blood glucose levels in incidentally discovered, untreated diabetes. The patient was stabilized with catheterization and pharmacotherapy, and the departments of Cardiovascular Medicine and Diabetology and Endocrinology approved the resumption of chemotherapy, upon which the patient resumed the same therapy. Compared to initial administration, paronychia reached grade 3 but was allowed to continue for approximately two years with dermatological intervention during repeated exacerbations and improvements. After PD, the patient received the best supportive care and died a few months later. The change in side effects between initial induction and reinduction is shown in Figure 3.

## Discussion

In the 2022 guidelines for the treatment of colorectal cancer, the chemotherapy algorithm for unresectable advanced or recurrent colorectal cancer does not list drug reuse except for Bev [5]. In clinical practice, however, occasionally there are cases in which the drugs in this algorithm are used up. In such cases, drugs that have shown a positive effect in the past or have controlled the disease with fewer side effects are often re-selected.

The Japan Clinical Cancer Research Organization (JACCRO) has reported positive effects of re-administration of anti-EGFR antibody drugs (Cmab: JACCRO-CC08 and Pmab: JACCRO-CC09) [6,10]; however, these reports only referred to side effects at the time of re-administration. The results of post-marketing surveillance of Cmab and Pmab have been reported in Japan, and it has been noted that Pmab-treated patients whose prior therapy included Cmab had a shorter time to the onset of hypomagnesemia and were more likely to develop severe hypomagnesemia [11,12]. This suggests the possibility of the early onset of side effects due to the increased cumulative dosage of anti-EGFR antibody drugs with the re-administration of these drugs. However, side effects other than those mentioned above are unclear regarding comparisons between the initial and reintroduced administrations.

This study focused on the side effects during initial and reintroduced administration, including hypomagnesemia. However, the effect of concomitant medications, such as proton pump inhibitors and loop diuretics, which have been previously reported [13,14], could not be examined in this study. This should be part of future studies to identify the risk factors.

Although there is much unclear regarding the possible mechanisms, several theories have been proposed regarding skin symptoms: (i) induction of keratinization abnormalities and inflammation due to disruption of epidermal turnover, (ii) increased production of inflammatory cytokines (e.g., IL-1, IL-6, TNF-α), and (iii) breakdown of immune barrier function [15]. Furthermore, regarding hypomagnesemia, a link has been suggested with the transient receptor potential melastatin (TRPM) 6 cation channel present in the distal tubules of the kidneys, and it has been reported that EGFR inhibitors cause downregulation of TRPM6 [16]. TRPM6 has genetic polymorphisms that have been reported to cause familial hypomagnesemia [17]. However, the association with increased side effects upon re-administration is unclear, and this, along with skin symptoms, remains a topic for future study.

From the perspective of drug administration guidance, patients should be informed about the pharmacological and side effects at the time of initial administration, with a special emphasis on side effect management at the time of re-administration. With reference to the report by Yamamoto et al. and the guide for appropriate use, medical professionals are paying attention to managing the side effects of anti-EGFR antibody drugs in the early stages of their use [18]. Therefore, early intervention and patient education regarding its adverse effects are essential.

## Conclusions

Here, we reported three cases with enhanced side effects due to the re-administration of anti-EGFR antibody drugs. The findings show that in some cases, the same side effects occur more intensely at reintroduction than at initial introduction, while in other cases, different side effects occur earlier. There is an urgent need for multidisciplinary involvement in the management of side effects, including skin disorders, for the continuation of treatment while preventing serious complications. We believe that repeated patient education is essential for this purpose.
